# A molecular model for self-assembly of the synaptonemal complex protein SYCE3

**DOI:** 10.1074/jbc.RA119.008404

**Published:** 2019-04-25

**Authors:** Orla M. Dunne, Owen R. Davies

**Affiliations:** From the Institute for Cell and Molecular Biosciences, Faculty of Medical Sciences, Newcastle University, Framlington Place, Newcastle upon Tyne NE2 4HH, United Kingdom

**Keywords:** meiosis, protein self-assembly, small-angle X-ray scattering (SAXS), molecular modeling, biophysics, structural biology, chromatin structure, coiled-coil, domain swap, SYCE3, synaptonemal complex, chromosomes, protein structure

## Abstract

The synaptonemal complex (SC) is a supramolecular protein assembly that mediates homologous chromosome synapsis during meiosis. This zipper-like structure assembles in a continuous manner between homologous chromosome axes, enforcing a 100-nm separation along their entire length and providing the necessary three-dimensional framework for cross-over formation. The mammalian SC comprises eight components—synaptonemal complex protein 1–3 (SYCP1–3), synaptonemal complex central element protein 1–3 (SYCE1–3), testis-expressed 12 (TEX12), and six6 opposite strand transcript 1 (SIX6OS1)—arranged in transverse and longitudinal structures. These largely α-helical, coiled-coil proteins undergo heterotypic interactions, coupled with recursive self-assembly of SYCP1, SYCE2–TEX12, and SYCP2–SYCP3, to achieve the vast supramolecular SC structure. Here, we report a novel self-assembly mechanism of the SC central element component SYCE3, identified through multi-angle light scattering and small-angle X-ray scattering (SAXS) experiments. These analyses revealed that SYCE3 adopts a dimeric four-helical bundle structure that acts as the building block for concentration-dependent self-assembly into a series of discrete higher-order oligomers. We observed that this is achieved through staggered lateral interactions between self-assembly surfaces of SYCE3 dimers and through end-on interactions that likely occur through intermolecular domain swapping between dimer folds. These mechanisms are combined to achieve potentially limitless SYCE3 assembly, particularly favoring formation of dodecamers of three laterally associated end-on tetramers. Our findings extend the family of self-assembling proteins within the SC and reveal additional means for structural stabilization of the SC central element.

## Introduction

**Figure 1.**
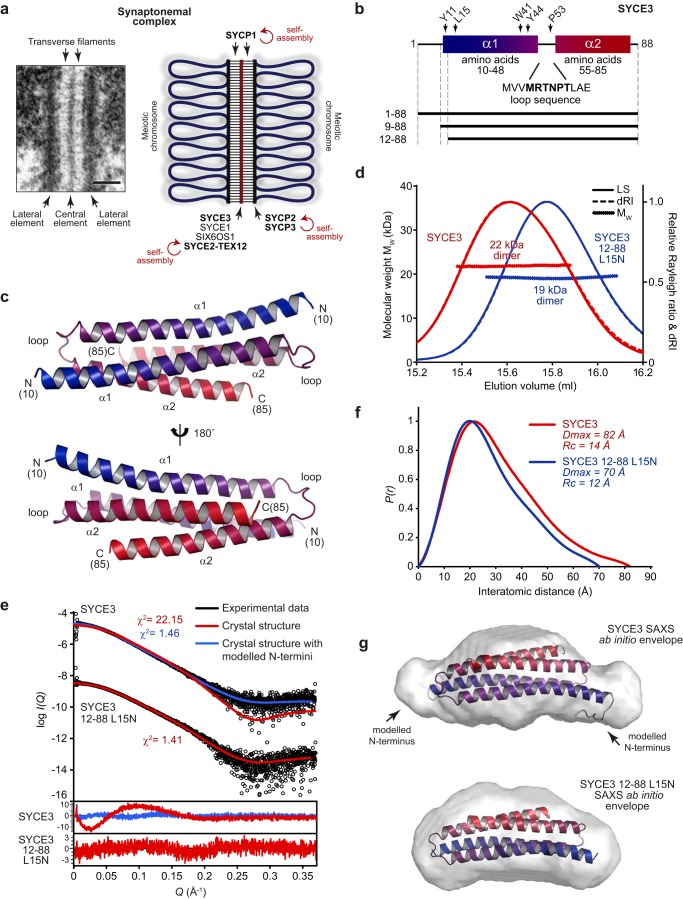
**SYCE3 forms a compact dimeric structure in solution.** *a*, the SC, visualized by EM (*left*; reproduced from Ref. 6) and displayed as a schematic (*right*). *Scale bar*, 100 nm. A role for self-assembly in SC structure has been demonstrated for transverse filament protein SYCP1 (23), lateral element protein SYCP3 (28) and its complex with SYCP2 (30), and central element complex SYCE2–TEX12 (25). *b*, *schematic* of the SYCE3 sequence highlighting its two α-helices, corresponding to amino acids 10–48 and 55–85, and intervening loop sequence. The location of key residues and the principal constructs used in this study are indicated. *c*, crystal structure of mouse SYCE3, rebuilt and re-refined using deposited structure factors (PDB code 4R3Q (31)) to include loops and short N-terminal extensions that were not included in the original build. SYCE3 adopts a compact dimeric anti-parallel four-helical bundle structure formed through the intertwined assembly of two helix-loop-helix chains. SYCE3 chains are *colored blue* (N termini) to *red* (C termini). *d*, SEC-MALS analysis; light scattering (*LS*) and differential refractive index (*dRI*) profiles are overlaid, with fitted molecular weights (*M_W_*) plotted as *diamonds* across elution peaks. Human SYCE3 full-length (*red*) and 12–88 L15N (*blue*) form dimers of 22 and 19 kDa, respectively (theoretical dimer masses, 21 and 20 kDa). *e–g*, SEC-SAXS analysis. *e*, SAXS scattering data of SYCE3 full-length and 12–88 L15N overlaid with theoretical scattering curves of the crystal structure alone (*red*, χ^2^ = 22.15 and 1.41, respectively) and upon the inclusion of modeled N and C termini (*blue*, χ^2^ = 1.46 for full-length SYCE3). Residuals for each fit are shown (*inset*). *f*, SAXS *P*(*r*) interatomic distance distributions of SYCE3 full-length (*red*) and 12–88 L15N (*blue*), showing maximum dimensions of 82 and 70 Å, respectively. Their cross-sectional radii (*R_c_*) were determined as 14 and 12 Å, respectively (Fig. S1*f*). *g*, SAXS *ab initio* models of SYCE3 full-length (*top*) and 12–88 L15N (*bottom*); filtered averaged models were generated from 30 independent DAMMIF runs imposing P2 symmetry and docked with the crystal structure with and without modeled N and C termini, respectively. SYCE3 full-length: normalized spatial discrepancy (NSD) = 1.110 ± 0.236 and reference model χ^2^ = 1.13; 12–88 L15N: NSD = 1.005 ± 0.365 and reference model χ^2^ = 1.51.

The synaptonemal complex (SC)^2^ is a unique biological structure, formed through the supramolecular assembly of largely α-helical coiled-coil protein components, that binds together homologous chromosome pairs during the first meiotic division (1–3). The SC was first identified in meiotic cells through its characteristic electron microscopic appearance, in which a ribbon-like structure of three parallel electron-dense components provides a continuous, regular, and complete synapsis between parallel homologous chromosome axes (Fig. 1*a*) (1, 4). Its electron-dense components are described as a midline central element and two flanking lateral elements, with each lateral element assembled on one chromosome axis of the homologous pair. These structures are held together through a series of interdigitated transverse filaments, which enforce a separation of ∼100 nm between lateral elements and thereby between homologous chromosomes (5). In addition to mediating synapsis, the three-dimensional structure of the SC modifies meiotic chromosome structure and provides the necessary physical framework for the formation of genetic cross-overs that ensure the faithful segregation of homologues (6–8). Accordingly, structural integrity of the SC is essential for meiotic cell division and fertility in mice (6), and SC defects are associated with human cases of infertility, recurrent miscarriage, and aneuploidy (9–11).

In mammals, the SC is formed of at least eight protein components: SYCP1–3, SYCE1–3, TEX12, and SIX6OS1 (Fig. 1*a*) (12–19). SYCP1 provides transverse filaments by forming a supramolecular lattice between homologous chromosomes, in which SYCP1 molecules are bioriented with their N and C termini localized within central and lateral elements, respectively (12, 20–23). The SYCP1 lattice must be reinforced and extended by two classes of central element proteins to achieve the structural and functional maturation of the SC. First, synapsis initiation factors SYCE3, SYCE1, and SIX6OS1 are required to achieve short stretches of synapsis between chromosome axes through a proposed role in providing vertical and transverse structural supports to a nascent SYCP1 lattice (16, 18, 19, 23, 24). Second, synapsis elongation complex SYCE2–TEX12 is required for the completion of a single continuous synapsis between homologues, which may be achieved by providing longitudinal structural support for the SYCP1 lattice along the length of the central element (15, 17, 25, 26). SC lateral elements are formed by SYCP2 and SYCP3, which contribute to meiotic chromosome compaction by stabilizing the regular linear array of chromatin loops (13, 14, 27–29).

An emerging theme in SC protein biochemistry is the formation of obligate α-helical dimeric and tetrameric coiled-coils that self-assemble into supramolecular structures through sequences at their α-helical termini. SYCP1 is an elongated coiled-coil with a tetrameric interface at its N terminus that extends into two C-terminal coiled-coil dimers (23). Its N termini form head-to-head dimers-of-dimers that combine with tetramer interfaces to achieve a staggered lattice, whereas C termini undergo protonation-induced assembly into back-to-back tetramers (23). SYCE2–TEX12 is an α-helical 4:4 complex that self-assembles into long fibers with dimensions similar to those of the central element (25). SYCP3 is a tetrameric coiled-coil that self-assembles through N- and C-terminal sites into filaments with regular 23-nm striations that match the length of individual molecules (27–29). A similar repeating unit has also been observed in limited assemblies of an SYCP2–SYCP3 complex (30). Thus, whereas SYCP1 forms discrete interactions in lattice assembly, certain central and lateral element components self-assemble into elongated linear structures with the capacity to extend along the chromosome length (Fig. 1*a*).

SYCE3 is a small protein of 88 amino acids that is essential for SC assembly, meiotic division, and fertility (18). Its disruption in mice leads to failure of synapsis, with formation of only short discontinuous stretches of SYCP1 between homologues and failure of recruitment of other central element proteins (18, 19). Further, SYCE3 has been reported to interact with SYCP1 and SYCE1 through pulldown, co-immunoprecipitation, and the ability of SYCE3 to recruit SYCE1 to SYCP1 cytoplasmic aggregates in somatic cells (18, 31, 32). Thus, the role of SYCE3 as a synapsis initiation factor may be achieved by mediating SC central element interactions. The crystal structure of mouse SYCE3 has been reported, revealing a compact four-helical bundle formed of two helix-loop-helix chains interacting in an anti-parallel configuration (Fig. 1, *b* and *c*) (31). In addition to the dimeric structure, a series of higher-molecular weight SYCE3 oligomers were detected in solution through chemical cross-linking analysis (31). These observations suggested to us that, similar to other SC proteins, the SYCE3 α-helical dimer may undergo self-assembly into structures that contribute to SC architecture.

Here, we report a molecular mechanism for SYCE3 self-assembly through multi-angle light scattering and small-angle X-ray scattering studies combined with experimentally directed molecular modeling. We find that SYCE3 dimers readily self-assemble in solution through lateral interactions mediated by surface-exposed aromatic residues and end-on assembly through a potential intermolecular domain-swap event. These self-assembly mechanisms combine to achieve a stable dodecamer of three laterally associated end-on tetramers, which may further assemble into larger molecular species.

## Results

### SYCE3 adopts a compact dimeric four-helical structure

The previously reported SYCE3 crystal structure revealed a compact four-helical bundle formed of two intertwined helix-loop-helix chains in an anti-parallel configuration (Fig. 1, *b* and *c*) (PDB code 4R3Q (31)). Whereas the two loops had not been built into the deposited structure, we found that they were visible in electron density maps, so we rebuilt and re-refined the SYCE3 structure against the deposited experimental data (Fig. S1 (*a* and *b*) and Table 1). The resultant structure is more complete and covers amino acids 10–85, which includes the additional loops and short N-terminal extensions (Fig. 1*c*). This rebuilt SYCE3 structure was used in all subsequent analyses.

**Table 1:** **Data collection, phasing, and refinement statistics**

	SYCE3 (previously released structure), PDB code 4R3Q	SYCE3 (rebuilt/re-refined structure),*^a^* PDB code 6H86
**Data collection**		
Space group	R3	
Cell dimensions		
*a*, *b*, *c* (Å)	75.58, 75.58, 101.51	
α, β, γ (degrees)	90, 90, 120	
Wavelength	0.9796 Å	
Resolution (Å)	50–1.90 (1.93–1.90)*^b^*	
*R*_sym_	0.059 (0.486)	
Completeness (%)	97.1 (81.9)	
*I*/σ(*I*)	22.0 (1.8)	
Redundancy	3.9 (2.8)	
**Refinement**		
Resolution (Å)	23.66–1.90	23.66–1.90
No. of reflections	16,431	16,425
*R*_work_/*R*_free_	0.2147/0.2345	0.1922/0.2167
No. of atoms	1243	1389
Protein	1173	1316
Ligand/ion	0	0
Water	70	73
*B*-factors	45.65	58.51
Protein	45.11	58.72
Ligand/ion	NA*^c^*	NA
Water	54.79	54.78
Root mean square deviations		
Bond lengths (Å)	0.007	0.016
Bond angles (degrees)	0.874	1.232

*^a^* Rebuilt and re-refined from a previously released structure (PDB code 4R3Q) (31).

*^b^* Values in parentheses are for highest-resolution shell.

*^c^* NA, not applicable.

We first tested whether the solution structure of SYCE3 corresponds to its compact crystal structure. We utilized size-exclusion chromatography multi-angle light scattering (SEC-MALS) to determine the unambiguous molecular weight of species within a protein sample. SEC-MALS confirmed that SYCE3 forms a 22-kDa homodimer (Fig. 1*d* and Fig. S1*c*), whereas CD spectroscopy confirmed the presence of almost entirely α-helical structure (Fig. S1*d*). We analyzed the size and shape of proteins in solution by size-exclusion chromatography small-angle X-ray scattering (SEC-SAXS; Table 2). The SAXS scattering curve was poorly fitted by the crystal structure, with a χ^2^ value of 22.2 (Fig. 1*e* and Fig. S1*e*). Further, the SAXS real-space *P*(*r*) interatomic distance distribution indicated a maximum dimension of 82 Å (Fig. 1*f*), which is ∼10 Å larger than the length of the crystal structure, and its *ab initio* dummy-atom model resembled the crystal structure with additional mass at either end of its long axis (Fig. 1*g*). We reasoned that this may be due to the 9-amino acid N termini that are present at either end of the molecule but are absent from the crystal structure owing to lack of electron density. We modeled N termini onto the structure using MODELLER, with them adopting largely flexible conformations (Fig. 1*g*). This full-length model fitted closely to experimental SAXS data with a χ^2^ value of 1.46 (Fig. 1*e*), indicating that scattering data are well-explained by the crystal structure with additional unstructured N termini. To confirm these findings, we analyzed a truncated construct of amino acids 12–88 (with subsequently described point mutation L15N), in which unstructured N termini are removed. SEC-MALS and CD analysis confirmed that this construct forms an α-helical dimer in solution (Fig. 1*d* and Fig. S1*d*). Further, its SEC-SAXS scattering data were closely fitted by the crystal structure (χ^2^ = 1.41) (Fig. 1*e*), with the *P*(*r*) distribution demonstrating a maximum dimension of 70 Å (Fig. 1*f*), and its *ab initio* model closely matching the crystal structure (Fig. 1*g*). Finally, SYCE3 full-length and 12–88 L15N demonstrated similar SAXS cross-sectional radii (Fig. S1*f*), in keeping with the presence of a common structural core. Thus, we conclude that solution-state SYCE3 adopts the compact dimeric fold observed in its crystal structure.

**Table 2:** **Summary of biophysical data**

	SYCE3 1–88				SYCE3 1–88 W41E/Y44E	SYCE3 1–88 W41A/Y44A		SYCE3 1–88 P53Q					SYCE3 1–88 PPP-loop	SYCE3 12–88 L15N
**SEC-MALS**														
Theoretical monomer weight (kDa)	10.7	10.7	10.7	10.7	10.7	10.5	10.5	10.8	10.8	10.8	10.8	10.8	10.6	10.3
Experimental molecular weight (kDa)	21.9	42.0	62.3	131.9	20.9	20.3	39.5	High-molecular weight assemblies					20.2	19.1
Oligomer	Dimer	Tetramer	Hexamer	Dodecamer	Dimer	Dimer	Tetramer	Assembly 1	Assembly 2	Assembly 3	Assembly 4	Assembly 5	Dimer	Dimer
**SEC-SAXS**														
*I*(0) (cm^−1^) (from *P*(*r*))	9.28 × 10^−3^ ± 9.57 × 10^−6^	8.34 × 10^−3^ ± 2.72 × 10^−5^	9.84 × 10^−3^ ± 3.55 × 10^−5^	2.94 × 10^−2^ ± 5.08 × 10^−5^	8.79 × 10^−3^ ± 2.28 × 10^−5^	6.03 × 10^−3^ ± 3.65 × 10^−5^	1.65 × 10^−2^ ± 7.31 × 10^−5^	4.12 × 10^−3^ ± 4.73 × 10^−5^	1.50 × 10^−2^ ± 5.80 × 10^−5^	2.17 × 10^−2^ ± 1.07 × 10^−4^	3.00 × 10^−2^ ± 1.65 × 10^−4^	3.82 × 10^−2^ ± 2.32 × 10^−4^	1.12 × 10^−2^ ± 1.73 × 10^−5^	3.11 × 10^−2^ ± 3.24 × 10^−5^
*I*(0) (cm^−1^) (from Guinier analysis*)*	9.20 × 10^−3^ ± 1.40 × 10^−5^	8.20 × 10^−3^ ± 2.10 × 10^−5^	9.80 × 10^−3^ ± 3.20 × 10^−5^	2.90 × 10^−2^ ± 6.60 × 10^−5^	8.80 × 10^−3^ ± 1.90 × 10^−5^	6.01 × 10^−3^ ± 3.60 × 10^−5^	1.60 × 10^−2^ ± 6.50 × 10^−5^	4.20 × 10^−3^ ± 5.80 × 10^−5^	1.50 × 10^−2^ ± 6.50 × 10^−5^	2.10 × 10^−2^ ± 1.20 × 10^−4^	3.00 × 10^−2^ ± 2.00 × 10^−4^	3.80 × 10^−2^ ± 3.40 × 10^−4^	1.10 × 10^−2^ ± 2.20 × 10^−5^	3.10 × 10^−2^ ± 3.30 × 10^−5^
*R_g_* (Å) (from *P*(*r*))	24.2 ± 0.51	31.1 ± 0.19	35.5 ± 0.13	44.9 ± 0.09	23.0 ± 0.08	23.6 ± 0.21	39.4 ± 0.24	47.8 ± 0.63	55.2 ± 0.24	68.6 ± 0.37	84.2 ± 0.51	103.8 ± 0.63	22.5 ± 0.06	21.4 ± 0.03
*R_g_* (Å) (from Guinier analysis)	23.5 ± 0.07	29.3 ± 2.79	34.7 ± 3.93	44.3 ± 4.38	22.5 ± 1.88	22.9 ± 0.23	36.8 ± 0.25	47.2 ± 4.23	53.6 ± 1.21	64.7 ± 3.62	81.3 ± 4.33	99.5 ± 5.84	22.2 ± 0.08	21.1 ± 1.70
*R_c_* (Å)	13.9	16.0	18.7	27.1	13.3	12.3	12.1	21.4	24.6	26.6	30.7	40.2	13.4	12.2
*D*_max_ (Å)	82	118	135	165	75	80	136	165	180	235	285	350	72	70
Porod volume (Å^3^)	40,399	66,227	10,1023	187,789	38,438	42,327	77,089	245,045	333,449	481,348	673,510	1,017,693	42,845	31,936
Molecular weight (from Porod volume) (kDa)	23.8	39.0	59.4	110.5	22.6	24.9	45.4	144.1	196.2	283.2	396.2	598.6	25.2	18.8
Volume of correlation (*V_c_*) (Å^2^)	257.9	364.8	463.0	785.6	246.5	238.6	405.9	720.1	970.4	1236.0	1715.4	2467.3	247.3	224.5
Molecular weight (from *V_c_*) (kDa)	23.0	36.9	50.3	113.1	21.9	20.2	36.4	89.3	142.7	191.8	294.2	497.3	22.4	19.4
*Ab initio* model fit (χ^2^)	1.13	NA*^a^*	NA	NA	1.21	1.12	1.28	NA	NA	NA	NA	NA	1.26	1.51
CRYSOL crystal structure fit (χ^2^)	22.15	NA	NA	NA	2.19	1.15	NA	NA	NA	NA	NA	NA	2.78	1.41
CRYSOL modeled structure fit (χ^2^)	1.46	4.95	14.47	NA	1.24	NA	1.18	NA	NA	NA	NA	NA	1.44	NA
CORAL rigid body fit (χ^2^)	NA	1.49	1.19	1.00	NA	NA	NA	NA	NA	NA	NA	NA	NA	NA
MONSA multiphase *ab initio* model fit (χ^2^)	1.23–1.25	1.10	1.12	1.73	NA	1.16–1.17	1.16	NA	NA	NA	NA	NA	NA	NA

*^a^* NA, not applicable.

### SYCE3 dimers undergo self-assembly into higher-order oligomers

**Figure 2.**
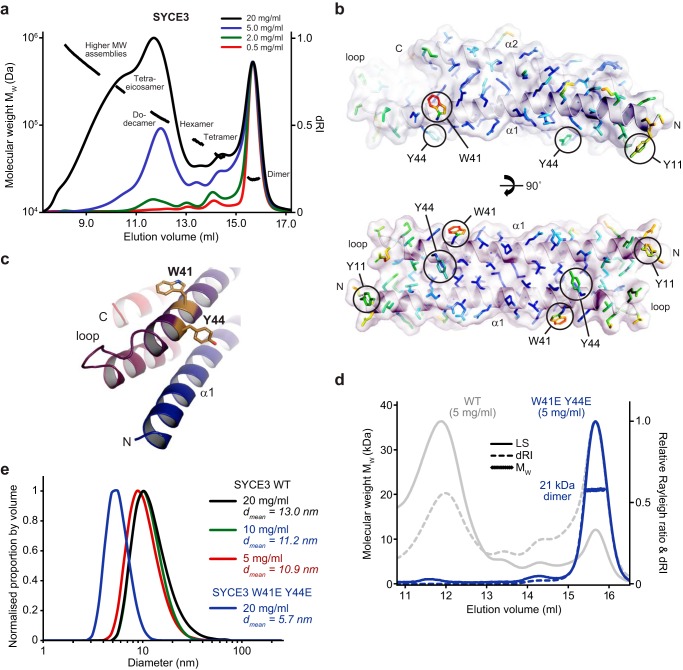
**SYCE3 self-assembly into higher-order oligomers.** *a*, SEC-MALS analysis of an SYCE3 concentration series (as indicated), plotted as differential refractive index (dRI) normalized to a constant dimer peak height, alongside molecular weight (*M_W_*). SYCE3 forms a discrete series of oligomers (dimer, tetramer, hexamer, dodecamer, and tetraeicosomer) that were confirmed through analysis of enriched species and fusion proteins (Fig. S2, *a–c*). *b*, molecular structure of SYCE3 with amino acid side chains *colored* according to their crystallographic *B*-factors from *red* (high) to *blue* (low). High *B*-factors were observed for solvent-exposed aromatic side chains Trp-41, Tyr-44, and Tyr-11, of which residues from both chains lie on the same surface of the molecule. *c*, Trp-41 and Tyr-44 residues are located in close proximity, toward the loop-end of the α1 helix. *d*, SEC-MALS analysis. SYCE3 W41E/Y44E (*blue*) is a 21-kDa dimer (theoretical dimer, 21 kDa) that fails to undergo higher order assembly; WT is shown in *gray* for comparison. *e*, DLS analysis displayed as normalized size distribution by volume, with mean particle diameter (*d*_mean_) indicated. WT SYCE3 forms large particles that increase in size and proportion with concentration, whereas W41E/Y44E forms only small particles of mean diameter 5.7 nm.

We next studied the ability of SYCE3 to undergo self-assembly in solution. SEC-MALS analysis revealed a concentration-dependent assembly of SYCE3 into a series of discrete higher-molecular weight species (Fig. 2*a*), with the proportion of assembled (nondimeric) material increasing from 20 to 85% (by mass) between concentrations of 0.5 and 20 mg/ml. The oligomer status of the SYCE3 assembly series was confirmed through SEC-MALS analysis of enriched samples as tetramer (42 kDa), hexamer (62 kDa), dodecamer (132 kDa), and tetraeicosamer (264 kDa) (Fig. 2*a* and Fig. S2 (*a* and *b*)). Further, MBP-SYCE3 fusion protein also assembled into tetramers and hexamers (Fig. S2*c*). The identification of these discrete molecular assemblies, and lack of intermediate oligomers, indicates that SYCE3 self-assembly cannot be explained by the nonspecific aggregation of SYCE3 dimers. Instead, it likely occurs through specific protein–protein interfaces that permit the formation of only a defined subset of oligomers and favor certain assemblies such as the dodecamer, which is clearly enriched across the concentration series (Fig. 2*a*).

We reasoned that SYCE3 self-assembly interactions are likely mediated by bulky surface-exposed residues that do not contribute to the dimeric structure. We identified Tyr-11, Trp-41, and Trp-44 as surface aromatic residues that exhibit high crystallographic *B*-factors (Fig. 2*b*), indicating that their side chains are flexible and do not contribute to the dimer fold. The proximity of Trp-41 and Tyr-44 residues at the end of the first α-helix, immediately prior to the loop, suggested that they may contribute to the same self-assembly interface (Fig. 2*c*). Accordingly, mutation of Trp-41 and Tyr-44 residues to glutamate completely inhibited SYCE3 self-assembly, as determined by SEC-MALS, leaving a nonassembled 21-kDa dimer (Fig. 2*d*). We verified that SYCE3 W41E/Y44E adopts the same compact dimeric structure as the WT protein through CD and SEC-SAXS analysis (Figs. S1*d* and S3 (*a–d*)).

To confirm our findings, we performed dynamic light scattering (DLS) analysis of total protein samples. SYCE3 WT formed wide populations of large-molecular weight species, with increased positive skew and rise in mean particle diameter from 10.9 to 13.0 nm between concentrations of 5 and 20 mg/ml (Fig. 2*e*). In contrast, SYCE3 W41E/Y44E at 20 mg/ml gave a narrow distribution with a mean particle diameter of 5.7 nm (Fig. 1*e*), in keeping with the 6.8-nm diameter of the SYCE3 crystal structure. We conclude that SYCE3 undergoes self-assembly through specific protein–protein interfaces, including one formed by Trp-41 and Tyr-44 aromatic side chains.

### Assembly of an elongated end-on tetramer by SYCE3 W41A/Y44A

**Figure 3.**
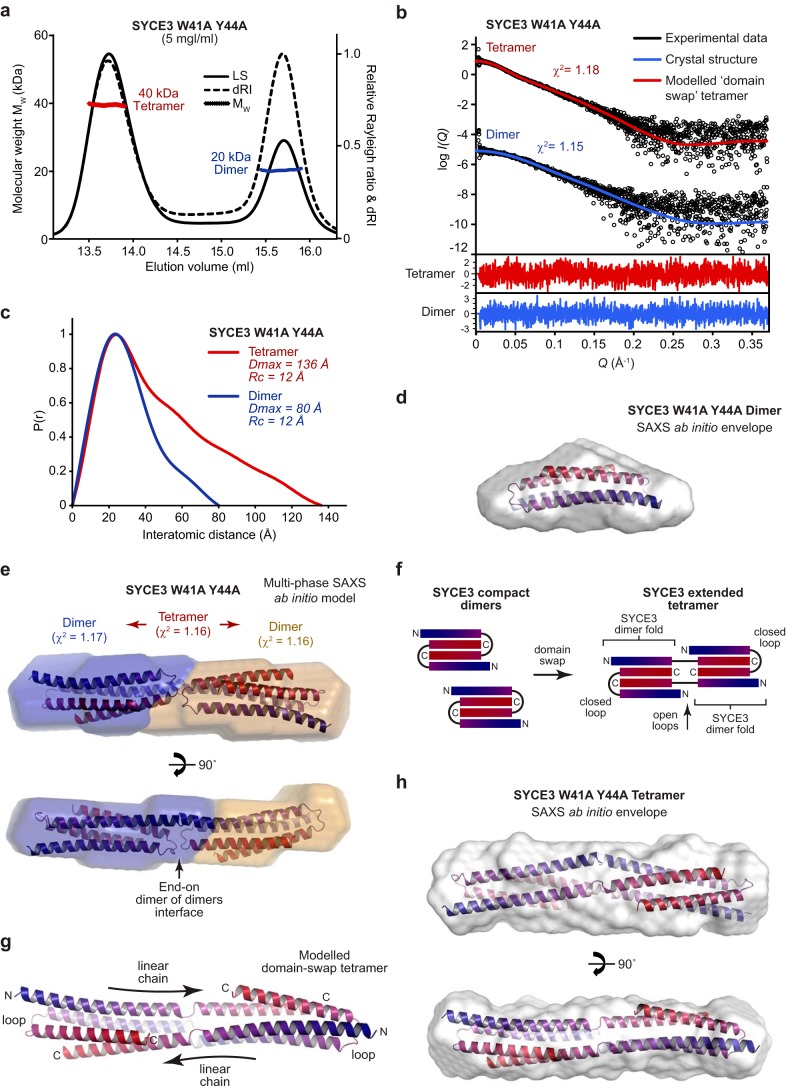
**SYCE3 W41A/Y44A forms an elongated end-on tetramer.** *a*, SEC-MALS analysis. SYCE3 W41A/Y44A forms approximately equal quantities (by mass) of a 20-kDa dimer and 40-kDa tetramer (theoretical masses, 21 and 43 kDa). *b–e*, SEC-SAXS analysis of SYCE3 W41A/Y44A dimer and tetramer species. *b*, SAXS scattering data of the W41A/Y44A dimer and tetramer overlaid with theoretical scattering curves of the SYCE3 crystal structure (*blue*, χ^2^ = 1.15) and a modeled domain-swap tetramer (*red*, χ^2^ = 1.18), respectively. Residuals for each fit are shown (*inset*). *c*, SAXS *P*(*r*) interatomic distance distributions of the W41A/Y44A dimer (*blue*) and tetramer (*red*), showing maximum dimensions of 80 and 136 Å, respectively. Their cross-sectional radii (*R_c_*) are both 12 Å (Fig. S3*h*). *d*, SAXS *ab initio* model of the W41A/Y44A dimer. A filtered averaged model was generated from 30 independent DAMMIF runs imposing P2 symmetry, with an NSD value of 1.293 ± 0.284 and reference model χ^2^ value of 1.12, and docked with the SYCE3 crystal structure. *e*, multiphase SAXS *ab initio* (MONSA) model of the W41A/Y44A tetramer (entire envelope; χ^2^ = 1.16), consisting of two W41A/Y44A dimers (*blue* and *yellow envelopes*; χ^2^ = 1.17 and 1.16), with the SYCE3 crystal structure docked into each of the dimer envelopes. *f*, *schematic* of proposed domain-swap tetramer assembly by SYCE3 in which two compact dimers interact through exchange of one α1 helix, coupled with loop opening, to form an elongated tetramer of two consecutive SYCE3 dimer folds. *g*, molecular model of an SYCE3 domain-swap tetramer generated by constrained docking of two rigid-body SYCE3 crystal structures, connected through flexible loop sequences, with subsequent energy minimization. The resultant structure consists of two linear SYCE3 chains of open loop conformation, with each dimer fold completed by capping SYCE3 chains of closed loop conformation. *h*, SAXS *ab initio* model of the W41A/Y44A tetramer. A filtered averaged model was generated from 30 independent DAMMIN runs imposing P2 symmetry, with an NSD value of 1.198 ± 0.667 and reference model χ^2^ value of 1.28 and docked with the modeled domain-swap tetramer.

Our analysis of the role of Trp-41 and Tyr-44 in SYCE3 self-assembly led to the observation that their individual alanine mutations stabilize a series of oligomeric species intermediates while blocking high-molecular weight assembly (Fig. S3, *e* and *f*). Further, SEC-MALS analysis of double-mutant W41A/Y44A revealed the presence of equal quantities (by mass) of dimers (20 kDa) and tetramers (40 kDa), with no higher-order oligomers (Fig. 3*a*). Thus, W41A/Y44A provided an important opportunity to analyze the structure of a tetrameric assembly intermediate.

We first confirmed that the W41A/Y44A dimer adopts the same structure as WT SYCE3. SEC-SAXS analysis determined a scattering curve that was closely fitted by the crystal structure (χ^2^ = 1.15) (Fig. 3*b* and Fig. S3*g*), with a *P*(*r*) distribution demonstrating a maximum dimension of 80 Å and an *ab initio* dummy-atom model that matches the crystal structure (Fig. 3, *c* and *d*). Thus, the compact dimer fold is unaffected in the W41A/Y44A mutant. We next performed SEC-SAXS analysis of the W41A/Y44A tetramer. Its *P*(*r*) distribution demonstrated a maximum dimension of 136 Å (Fig. 3 (*b* and *c*) and Fig. S3*g*), which is almost double the length of the dimer, whereas both dimers and tetramers had identical cross-sectional radii (Fig. S3*h*), suggesting that the tetramer may form through end-on interactions of SYCE3 dimers. As a complementary unbiased approach, we performed multiphase SAXS *ab initio* modeling of the SYCE3 W41A/Y44A tetramer to identify the relative position of its constituent dimers. This demonstrated a linear arrangement of dimer envelopes to create an elongated tetramer envelope, with docked SYCE3 dimer structures orientated with an apparent end-on self-assembly interface mediated by SYCE3 loops (Fig. 3*e*).

The helix-loop-helix conformation adopted by SYCE3 chains within the dimer fold suggested that its end-on interaction may be mediated by an intermolecular domain-swap event, rather than a simple noncovalent interaction between its loops. In this, we propose that interacting loops must open to create linear SYCE3 chains in which a helix from one dimer fold is swapped with the equivalent helix of its interacting dimer (Fig. 3*f*). We built a theoretical model of a domain-swap tetramer through rigid-body docking of dimer folds connected by flexible intermolecular loops, coupled with geometry and energy minimization, confirming that such a configuration is structurally plausible (Fig. 3*g*). The modeled domain-swap tetramer was fitted to the SAXS scattering curve of the W41A/Y44A tetramer with a χ^2^ value of 1.18 (Fig. 3*b*) and closely matched the dimensions of its SAXS *ab initio* molecular envelope (Fig. 3*h*). We conclude that the W41A/Y44A tetramer is formed through end-on interaction between dimers, which most likely occurs through a domain-swap event in which interacting loops open to create continuous SYCE3 chains that provide tight associations between dimer folds.

### An open loop conformation is required for SYCE3 self-assembly

**Figure 4.**
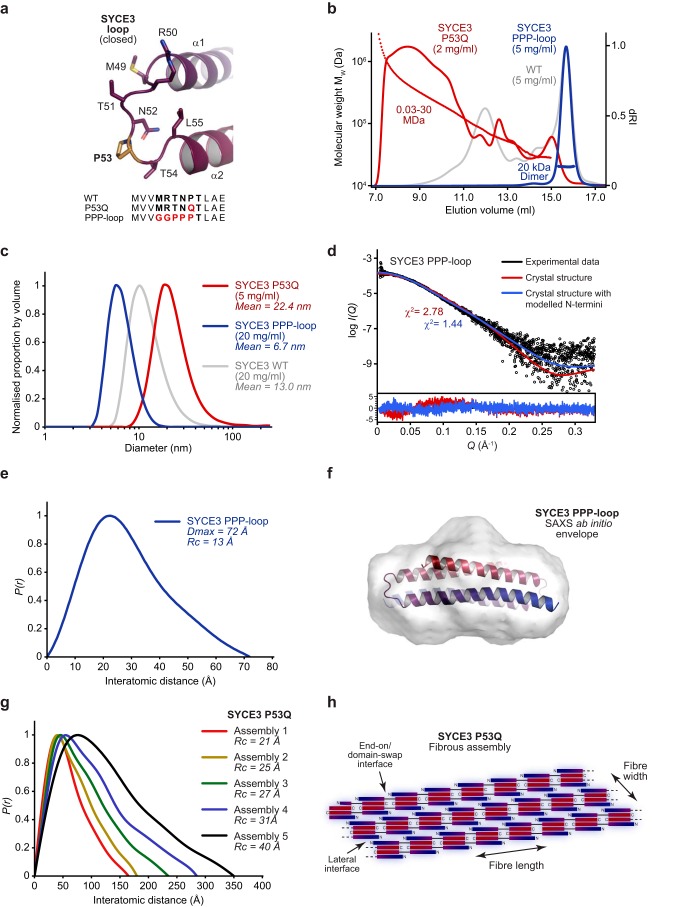
**SYCE3 self-assembly requires formation of an open loop conformation.** *a*, the SYCE3 loop adopts a closed conformation in the dimer structure, in which it provides a turn between anti-parallel α1 and α2 helices. Point mutation P53Q was designed to prevent loop closure and encourage formation of an open conformation, whereas the PPP-loop mutation was designed to retain the closed conformation and prevent loop-opening. *b*, SEC-MALS analysis. SYCE3 P53Q (*red*) fails to form dimers and instead assembles into large-molecular weight species, whereas PPP-loop (*blue*) blocks higher-order assembly, leaving a 20-kDa dimer (theoretical dimer, 21 kDa). WT is shown in *gray* for comparison. *c*, DLS analysis. SYCE3 P53Q (*red*) forms large particles of size greater than WT assemblies (*gray*), whereas PPP-loop (*blue*) forms only small particles of mean diameter 6.7 nm. *d–g*, SEC-SAXS analysis. *d*, SAXS scattering data of SYCE3 PPP-loop overlaid with the theoretical scattering curve of the SYCE3 crystal structure alone (red, χ^2^ = 2.78) and upon the inclusion of modeled N and C termini (*blue*, χ^2^ = 1.44). Residuals for each fit are shown (*inset*). *e*, SAXS *P*(*r*) interatomic distance distribution of SYCE3 PPP-loop, showing a maximum dimension of 72 Å. Its cross-sectional radius (*R_c_*) was determined as 13 Å (Fig. S4*b*). *f*, SAXS *ab initio* model of SYCE3 PPP-loop. A filtered averaged model was generated from 30 independent DAMMIF runs imposing P2 symmetry, with an NSD value of 1.392 ± 0.185 and reference model χ^2^ value of 1.26, and docked with the SYCE3 crystal structure. *g*, SAXS *P(r*) interatomic distance distributions of SYCE3 P53Q species (assemblies 1–5 represent the smallest to largest size-exclusion chromatography elution species), with cross-sectional radii indicated. *h*, model of SYCE3 P53Q self-assembly in which recursive domain-swap interactions provide the length of fibers, whereas their lateral associations provide width.

We next sought to determine whether SYCE3 self-assembly involves the domain-swap event proposed for the W41A/Y44A tetramer. We reasoned that domain swap must require a specific loop sequence that can accommodate both the tight turn of the closed conformation and the linear configuration of the open conformation. Specifically, the backbone geometry of proline residue Pro-53 may be important for the closed conformation, whereas surrounding amino acids may stabilize the open conformation (Fig. 4*a*). To test this, we designed mutation P53Q to block the closed conformation and replaced the loop with alternative sequence ^49^GGPPP^53^ (PPP-loop) to block the open conformation (Fig. 4*a*).

SEC-MALS analysis revealed that SYCE3 P53Q formed large-molecular weight assemblies and lacked dimeric species, in keeping with an inability for loop closure to support the compact dimer (Fig. 4*b*). In contrast, PPP-loop formed a 20-kDa dimer that failed to self-assemble into higher-molecular weight species (Fig. 4*b*). We confirmed these findings by DLS analysis. SYCE3 P53Q showed a wide distribution of large molecular weight species, whereas SYCE3 PPP-loop gave a narrow distribution with mean particle diameter of 6.7 nm, matching the diameter of the crystal structure (Fig. 4*c*). SEC-SAXS analysis of the PPP-loop dimer revealed a scattering curve, cross-sectional radius, *P*(*r*) distribution, and *ab initio* dummy-atom model that match the crystal structure, indicating that the loop mutations had successfully sustained the compact dimer (Fig. 4 (*d–f*) and Fig. S4 (*a* and *b*)). Whereas SYCE3 P53Q did not resolve into discrete oligomeric species, it was possible to perform SEC-SAXS analysis of five regions of its elution in which scattering data showed local consistency. The resultant *P*(*r*) distributions demonstrated a positive skew that increased with assembly size, between maximum dimensions of 165 and 350 Å, indicating rodlike structures of increasing length (Fig. 4*g* and Fig. S4 (*c* and *d*)). This is consistent with the P53Q mutation imposing a constitutively open loop conformation such that fibers of linear chains of SYCE3 dimer folds are formed through recursive end-on/domain-swap events (Fig. 4*h*). Further, the cross-sectional radius increased from 21 to 40 Å (Fig. S4*e*), indicating that growth of fiber length is coupled with an increase in width, possibly through lateral interactions between SYCE3 end-on/domain-swap chains mediated by native self-assembly interfaces (Fig. 4*h*). Finally, we confirmed that WT SYCE3 interacts with and is incorporated into P53Q assemblies, suggesting that the structures formed by P53Q reflect those of native self-assembly (Fig. S4*f*). Thus, through analysis of P53Q and PPP-loop mutants, we conclude a crucial role for the SYCE3 loop in self-assembly that is consistent with end-on interactions occurring through a domain-swap mechanism.

### The α1 N-terminal tip is required for SYCE3 self-assembly

**Figure 5.**
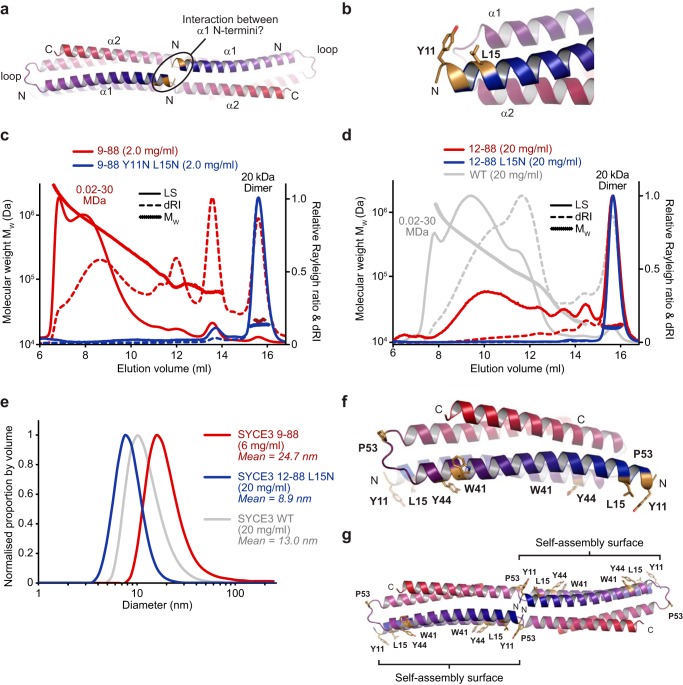
**SYCE3 self-assembly is facilitated by the N-terminal tip of the α1 helix.** *a*, SYCE3 modeled domain-swap tetramer in which the N termini of capping chain α1 helices are highlighted, indicating their potential proximity and possible interaction in assembled structures. *b*, the N terminus of α1 helix includes amino acids Tyr-11 and Leu-15, which are solvent-exposed and do not contribute to the SYCE3 dimer fold. *c* and *d*, SEC-MALS analysis. *c*, SYCE3 9–88 (*red*) forms high-molecular weight assemblies, whereas 9–88 Y11N/L15N (*blue*) is largely restricted to a 20-kDa dimer (theoretical dimer, 21 kDa). *d*, SYCE3 12–88 (*red*) is mostly dimeric with some higher-order structures, whereas 12–88 L15N (*blue*) forms only a 20-kDa dimer (theoretical dimer, 21 kDa); WT is shown in *gray* for comparison. *e*, DLS analysis. SYCE3 9–88 (*red*) forms large particles of size greater than WT assemblies (*gray*), whereas 12–88 L15N (*blue*) forms only small particles of mean diameter 8.9 nm. *f* and *g*, summary of the molecular determinants of SYCE3 self-assembly. Shown are SYCE3 dimeric crystal structure (*f*) and modeled domain-swap tetramer (*g*) highlighting the positions of surface aromatic residues Trp-41 and Tyr-44, loop residue Pro-53, and α1 N-terminal residues Tyr-11 and Leu-15.

The W41A/Y44A domain-swap tetramer model indicated the close proximity of the N-terminal tips of opposing α1 helices, raising the possibility of their involvement in stabilization of the end-on interaction and SYCE3 self-assembly (Fig. 5, *a* and *b*). The removal of the unstructured SYCE3 N terminus in construct 9–88 promoted its self-assembly, with the presence of 85% assembled material (by mass) at 2.0 mg/ml, compared with 50% for WT (Fig. 5*c*). Further truncation to remove the α1 N-terminal tip, while retaining the structural core, in construct 12–88 inhibited self-assembly of the 20-kDa dimer, with the presence of only 30% (by mass) assembled material at 20 mg/ml, compared with 85% for WT (Fig. 5*d*). The α1 N-terminal tip includes amino acids Tyr-11 and Leu-15, which are located on the same side of the helix with solvent-exposed side chains (Fig. 5*b*). Their mutation to glutamine in 9–88 largely inhibited self-assembly with the presence of 5% (by mass) assembled material at 2.0 mg/ml, and the single mutation L15N eliminated remaining self-assembly of 12–88, with the presence of <5% (by mass) assembled material at 20 mg/ml (Fig. 5, *c* and *d*). DLS analysis revealed a wide distribution of large-molecular weight species for SYCE3 9–88, with mean particle diameter of 24.7 nm (compared with 13.0 nm for WT), and a narrow distribution with mean particle diameter of 8.9 nm for SYCE3 12–88 L15N (Fig. 5*e*). We confirmed that the SYCE3 dimer fold is retained in the 12–88 L15N construct, as described previously (Fig. 1 (*d–g*) and Fig. S1 (*d* and *f*)). We conclude that amino acids Tyr-11 and Leu-15 of the α1 N-terminal tip provide a third assembly interface, which must combine with the Trp-41/Tyr-44 interface and end-on interaction to achieve SYCE3 self-assembly. The Tyr-11/Leu-15 and Trp-41/Tyr-44 interfaces from both chains are located on the same side of the SYCE3 dimer, indicating the presence of a single self-assembly surface (Fig. 5*f*). Further, domain swap would impose a 180° rotation on the constituent dimer folds, positioning self-assembly surfaces on either side of the tetramer (Fig. 5*g*). Thus, our proposed model for end-on interaction through domain-swap positions self-assembly surfaces in a manner compatible with self-assembly as a plane of laterally associated tetramers.

### A molecular model for SYCE3 self-assembly

**Figure 6.**
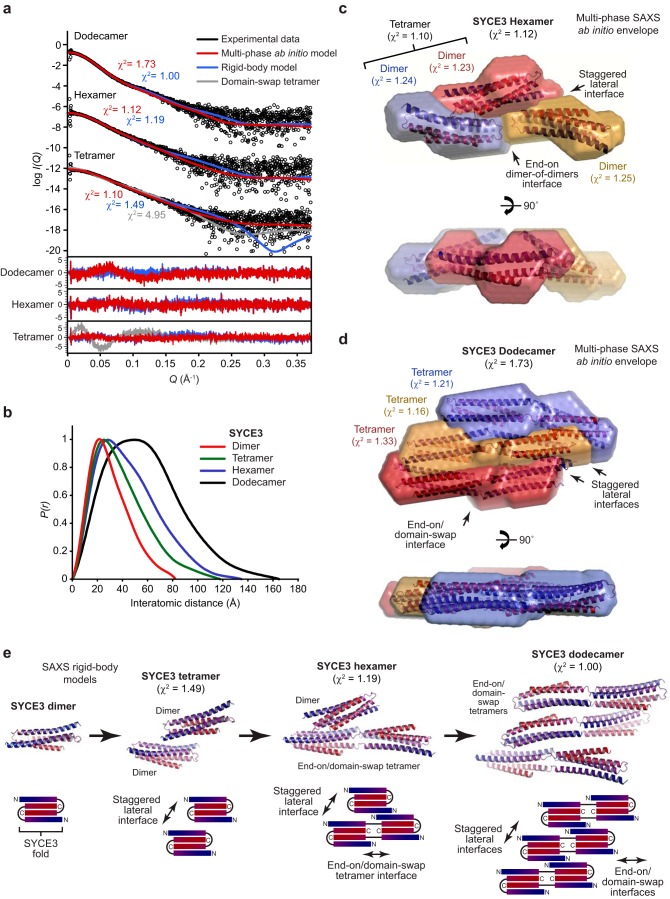
**Structural basis of SYCE3 self-assembly into higher order oligomers.** *a–e*, SEC-SAXS analysis of SYCE3 tetramer, hexamer, and dodecamer species. *a*, SAXS scattering data overlaid with the theoretical scattering curves of multiphase *ab initio* MONSA models (*red*), rigid-body CORAL models (*blue*), and the SYCE3 domain-swap hexamer (*gray*). χ^2^ values are indicated, and residuals for each fit are shown (*inset*). *b*, SAXS *P*(*r*) interatomic distance distributions of the SYCE3 dimer, tetramer, hexamer, and dodecamer, showing maximum dimensions of 82, 118, 135, and 165 Å, respectively. Their cross-sectional radii (*R_c_*) were determined as 14, 16, 19, and 27 Å, respectively (Fig. S5*b*). *c*, multiphase SAXS *ab initio* (MONSA) model of the SYCE3 hexamer (entire envelope; χ^2^ = 1.12), consisting of a tetramer (χ^2^ = 1.10) of two dimers (*blue* and *red envelopes*; χ^2^ = 1.24 and 1.23) and a third dimer (*yellow envelope*; χ^2^ = 1.25). The SYCE3 crystal structure was docked into each of the dimer envelopes. *d*, multiphase SAXS *ab initio* (MONSA) model of the SYCE3 dodecamer (entire envelope; χ^2^ = 1.73), consisting of three W41A/Y44A end-on tetramers (*blue*, *yellow*, and *red envelopes*; χ^2^ = 1.21, 1.16, and 1.33), with the modeled domain-swap tetramer docked into each envelope. *e*, SAXS rigid-body CORAL models of the SYCE3 tetramer formed of two dimers (χ^2^ = 1.49), hexamer of an end-on tetramer (modeled as a domain-swap tetramer) and a dimer (χ^2^ = 1.19), and a dodecamer of three modeled domain-swap tetramers (χ^2^ = 1.00). *Schematic diagrams* are included to illustrate the proposed mechanism of progressive assembly. The tetramer is formed of lateral associations between dimers, the hexamer consists of the same lateral association between a dimer and an end-on/domain-swap tetramer, and the dodecamer consists of lateral associations between three end-on/domain-swap tetramers.

We utilized SEC-SAXS analysis to establish the molecular nature of the SYCE3 tetramer, hexamer, and dodecamer self-assembly species (Fig. 6*a* and Fig. S5 (*a* and *b*)). The *P*(*r*) distributions of the tetramer and hexamer showed positive skew consistent with elongated molecules, whereas the dodecamer demonstrated a broad distribution, suggesting a flat particle (Fig. 6*b*). We performed multiphase SAXS *ab initio* modeling of the SYCE3 hexamer, using scattering data of the hexamer, tetramer, and dimer, to identify the relative position of SYCE3 dimers within the hexamer and constituent tetramer (Fig. 6*c*). This demonstrated that the tetramer consists of a staggered lateral association of two SYCE3 dimers (χ^2^ = 1.10), suggesting its formation through association between their self-assembly surfaces (Fig. 6*c*). We confirmed that the tetramer is not formed through domain swap through its poor fit to the scattering data (χ^2^ = 4.95; Fig. 6*a*). In the hexamer, the third dimer was positioned in an apparent end-on configuration with one component of the tetramer (χ^2^ = 1.12; Fig. 6*c*). Thus, the third dimer is recruited in an end-on manner, possibly through a domain-swap event, resulting in a hexamer in which an extended end-on tetramer is laterally associated with a dimer fold through interaction of their self-assembly surfaces.

The combination of end-on and lateral association in the SYCE3 hexamer suggested that the dodecamer may be formed of three laterally associated end-on tetramers. We performed multiphase SAXS *ab initio* modeling of the SYCE3 dodecamer, using scattering data of the dodecamer and W41A/Y44A tetramer, to identify the relative position of three constituent end-on tetramers. This demonstrated staggered lateral associations between end-on tetramers, with assembly largely in two dimensions, to generate a relatively flat dodecameric structure with a depth approximately equal to the diameter of an SYCE3 dimer (χ^2^ = 1.73; Fig. 6*d*). As a complementary approach, we performed SAXS rigid-body modeling of the tetramer, hexamer, and dodecamer utilizing the dimer and the domain-swap tetramer model (representing an end-on tetramer) as rigid bodies (Fig. 6*e*). The resultant tetramer and hexamer models demonstrated staggered lateral orientations between dimers and the dimer or domain-swap tetramer (χ^2^ = 1.49 and 1.19), and the dodecamer model demonstrated lateral interactions between domain-swap tetramers (χ^2^ = 1.00) (Fig. 6*e*). Thus, we propose that SYCE3 self-assembly is mediated by mutually reinforcing end-on and lateral interactions between Tyr-11/Leu-15 and Trp-41/Tyr-44 self-assembly interfaces. This model is readily explained by the orientation of SYCE3 self-assembly surfaces on either side of the domain-swap tetramer. Further, SYCE3 assemblies may be extended through further recursive end-on/domain-swap events and additional lateral associations, explaining the large fibrous assemblies observed for P53Q and the higher-molecular weight oligomers of WT SYCE3.

## Discussion

The SC is built from a small number of known protein components, with its elegant supramolecular assembly achieved through a combination of their discrete heterotypic interactions and recursive higher-order self-assembly. SC component self-assembly mechanisms have been previously reported for transverse filament protein SYCP1, central element complex SYCE2–TEX12, and lateral element components SYCP3 and an SYCP2–SYCP3 complex (23, 25, 27, 28, 30). Here, we extend this emerging theme by reporting a novel SC assembly mechanism through self-assembly of central element component SYCE3. We find that the dimeric SYCE3 building block assembles into a discrete series of oligomers in solution, including tetramer, hexamer, dodecamer, tetraeicosamer, and higher-order species. Analysis of WT and mutant oligomers, through SEC-MALS, SEC-SAXS, and SAXS-directed molecular modeling, revealed that hierarchical assembly is achieved through two mechanisms. First, SYCE3 dimers undergo a staggered lateral interaction between their self-assembly surfaces, which are formed by both copies of amino acids Trp-41 and Tyr-44 and α1 N-terminal tip residues Tyr-11 and Leu-15. Second, SYCE3 dimers undergo end-on interactions through their loop sequences. Whereas SAXS data cannot discriminate between end-on interaction modes, our mutagenesis and modeling data suggest that its likely mechanism is a domain-swap event in which a helix from one dimer is swapped with that of another, coupled with loop opening, to achieve an elongated tetramer consisting of two linear and two folded SYCE3 chains. The domain-swap tetramer positions self-assembly surfaces on opposing sides of the molecule, permitting staggered lateral interactions in both directions. Thus, the two assembly mechanisms are mutually reinforcing and collaborate to achieve a stable dodecamer of three laterally associated domain-swap tetramers. This structure can be extended, potentially indefinitely, through recursive end-on and lateral interactions, to achieve higher-order oligomers that may be stabilized within the context of the native SC.

What is the role of SYCE3 assembly within the SC structure? SYCE3, SYCE1, and SIX6OS1 have been described as synaptic initiation factors as their knockouts lead to a failure of SC tripartite structure formation, with the retention of only discontinuous SYCP1 assembly between homologues (16, 18, 19). On this basis, they have been proposed to provide short-range stabilization of the SYCP1 lattice, possibly in transverse or vertical planes (23). SYCE1 is a nonassembling dimer that may act as a strut within the central element (24), and SIX6OS1 has not yet been structural characterized, so SYCE3 constitutes the first reported self-assembling central element initiation factor. Synaptic elongation complex SYCE2–TEX12 is essential for extension of the SC tripartite structure, likely achieved through its self-assembly into long fibrous structures that provide longitudinal support of the SYCP1 lattice along the entire chromosome length (15, 17, 23, 25, 26). Thus, the relatively low-molecular weight SYCE3 assemblies, in contrast with micrometer-long SYCE2–TEX12 fibers (25), may reflect their respective roles in short range transverse/vertical and long-range longitudinal support of the SYCP1 lattice. The precise molecular connections of SYCE3 assemblies and their role in SYCP1 lattice stabilization must be elucidated through future structural analyses of their complexes with other SC central element components. These may include interactions with SYCP1 and SYCE1, which have been proposed on the basis of pulldown, co-immunoprecipitation, and co-recruitment to SYCP1 cytoplasmic aggregates (18, 31, 32).

SYCE3 self-assembly adds to the general theme of SC protein biochemistry in which coiled-coils assemble into supramolecular structures through sequences within their α-helical termini (23, 25, 28, 30). In this regard, SC proteins share similarities with intermediate filament proteins, such as lamins and keratins, that form the cytoskeleton (29, 33, 34). This family of proteins form extended α-helical coiled-coils, typically with nonhelical N and C termini, that assemble into higher-order structures (35). The range of self-assembly mechanisms reported for intermediate filaments bear similarities to those of SC protein assembly. These include lateral and end-on interactions of coiled-coils into assemblies and fibers, such as those observed in SYCE3 and SYCE2–TEX12 self-assembly (25). Further, intermediate filaments often demonstrate regular repeating units analogous to the 23-nm pattern of SYCP3 and SYCP2–SYCP3 assemblies (28, 30) and sometimes form paracrystalline arrays similar to SYCP3 filaments and SYCP1 polycomplexes (28, 29, 36). Despite these apparent structural and functional similarities, there is no overt amino acid sequence similarity between SC and intermediate filament proteins. Nevertheless, coiled-coil proteins frequently retain structure while exhibiting substantial sequence divergence. Indeed, SC transverse filament proteins in mammals, yeast, and *Drosophila* show no higher level of sequence similarity with each other than with unrelated coiled-coil proteins, despite their ability to form morphologically similar SC structures. Thus, it is intriguing to speculate whether the SC may have evolved through specific adaptations of intermediate filament proteins or whether the SC and intermediate filaments evolved separately but have found a common protein assembly mechanism through evolutionary convergence.

The role of protein self-assembly in the SC structure, which is of up to 24 μm in length and 100 nm in width and depth (1, 21, 37), follows the general principles and advantages of supramolecular structure formation through recursive protein interactions. In this, protein self-assembly achieves vast protein structures from a minimal genetic code, provides cooperativity between protomers, and enables simple mechanisms for assembly and disassembly through targeting of single interfaces (38–40). In the SC, structural changes within recursive assemblies may be transmitted cooperatively to propagate signals, such as those involved in cross-over interference, along the chromosome axis. Further, targeting of individual protein–protein and/or self-assembly interfaces through post-translational modifications, such as PLK1 phosphorylation of SYCP1 and TEX12 (41), may be sufficient to achieve the gradual but progressive disassembly of the SC supramolecular structure. In this regard, the paradigm of intermediate filament protein disassembly by phosphorylation *in vivo* may be directly applicable to SC proteins (35). Finally, in contrast to the purposeful self-assembly of proteins into the SC during meiosis, pathological protein self-assembly has been implicated in a number of distinct clinical conditions, including Alzheimer's disease, sickle cell anemia, cataracts formation, and Parkinson's disease (39, 40). Thus, in addition to elucidating the molecular basis of SC assembly, the general principles that emerge from this and other studies may contribute to our wider understanding of pathological protein self-assembly in human diseases.

## Experimental procedures

### Recombinant protein expression and purification

Human SYCE3 sequences harboring truncations and/or mutations (as indicated) were cloned into pMAT11 vectors (42) for bacterial expression as fusion proteins with tobacco etch virus–cleavable N-terminal His_6_-maltose-binding protein tags. For co-expression, His_6_-tagged SYCE3 was cloned into site two of pRSFDuet-1 vector (Novagen®). Proteins were expressed in the BL21(DE3) *Escherichia coli* cell line (Novagen®) and cultured in 2× YT medium. At optimum *A*_600 nm_, cells were induced with 0.5 mm isopropyl 1-thio-β-d-galactopyranoside at 25 °C for 16 h. Cells were lysed by sonication in 20 mm Tris, pH 8.0, 500 mm KCl. Fusion proteins were purified from the clarified lysate through nickel-nitrilotriacetic acid and amylose (New England Biolabs) affinity followed by HiTrap Q HP (GE Healthcare) anion-exchange chromatography. Tags were removed by incubation with tobacco etch virus protease, and cleaved protein was further purified by anion-exchange chromatography and, where appropriate, size-exclusion chromatography (HiLoad^TM^ 16/600 Superdex 200, GE Healthcare). Purified proteins were concentrated using Amicon Ultra® 10,000 molecular weight cutoff centrifugal filter units (Millipore), flash-frozen in liquid nitrogen, and stored at −80 °C. SDS-PAGE with Coomassie staining was used for protein sample analysis, and protein concentrations were determined using a Cary 60 UV spectrophotometer (Agilent) with molecular weights and extinction coefficients calculated using ExPASY ProtParam (http://web.expasy.org/protparam).

### CD spectroscopy

Far-UV CD spectra were measured using a Jasco J-810 spectropolarimeter (Institute for Cell and Molecular Biosciences, Newcastle University). Wavelength scans were carried out at 4 °C, between 260 and 185 nm, at 0.2-nm intervals using a 0.2-mm path length quartz cuvette (Hellma). Protein samples were at 0.2–0.4 mg/ml in 10 mm Na_2_HPO_4_, pH 7.5, 150 mm NaF. For each protein, nine measurements were recorded, averaged, buffer-corrected, and converted to mean residue ellipticity ([θ]) (×1000 degrees·cm^2^·dmol^−1^·residue^−1^). Deconvolution was performed using the Dichroweb CDSSTR algorithm (http://dichroweb.cryst.bbk.ac.uk)^3^ (55).

### SEC-MALS

The oligomeric state of SYCE3 constructs was determined by SEC-MALS. Protein samples were analyzed at 0.5–20 mg/ml in 20 mm Tris, pH 8.0, 150 mm KCl, 2 mm DTT. Samples were loaded at 0.5 ml/min onto a Superdex^TM^ 200 Increase 10/300 GL (GE Healthcare) column with an ÄKTA^TM^ Pure (GE Healthcare). The outflow from the column was fed into a DAWN® HELEOS^TM^ II MALS detector (Wyatt Technology), followed by an Optilab® T-rEX^TM^ differential refractometer (Wyatt Technology). ASTRA® 6 software (Wyatt Technology) was used to collect and analyze the data, using Zimm plot extrapolation with a *dn/dc* value of 0.185 ml/g to determine molecular weights from eluted protein peaks.

### SEC-SAXS

SEC-SAXS experiments were performed at Diamond Light Source synchrotron facility (Oxfordshire, UK) on the SAXS beamline B21. Protein samples at concentrations 5–20 mg/ml were loaded onto a Superdex^TM^ 200 Increase 10/300 GL size-exclusion chromatography column (GE Healthcare) in 20 mm Tris, pH 8.0, 150 mm KCl at 0.5 ml/min using an Agilent 1200 HPLC system. The column outflow passed through the experimental cell, where SAXS data were recorded at 12.4 keV, detector distance 4.014 m, in 3.0-s frames. ScÅtter version 3.0 (http://www.bioisis.net)^3^ (56) was used to subtract, average and carry out Guinier analysis for the *R_g_* and cross-sectional *R_g_* (*R_c_*); www.bayesapp.org^3^ was used to approximate parameters for real space analysis with final *P*(*r*) distributions fitted using PRIMUS (43). *Ab initio* modeling was performed using DAMMIF (44) or DAMMIN (45); 30 independent runs were performed in P2 symmetry and averaged. Multiphase SAXS *ab initio* modeling was performed using MONSA (45); rigid-body modeling was performed using CORAL (46). Crystal structures and models were docked into DAMFILT and MONSA molecular envelopes using SUPCOMB (47) and were fitted to experimental data using CRYSOL (48). Linear residuals are plotted as the difference between experimental and theoretical *I*(*Q*) values divided by experimental error.

### Rebuilding and re-refinement of the SYCE3 crystal structure

The previously determined crystal structure of SYCE3 (PDB code 4R3Q) (31) was rebuilt using the deposited experimental data, including the addition of four amino acid loops (residues 50–53) and three-amino acid N-terminal extensions (residues 10–12) to both chains. Model building was performed automatically using PHENIX Autobuild and PHENIX Fit Loops (49) and manually in Coot (50). The structure was refined using PHENIX refine (49) against data to 1.90 Å resolution, to *R* and *R*_free_ values of 0.1922 and 0.2167, respectively, with 98.65% of residues within the favored regions of the Ramachandran plot (0 outliers), clashscore of 3.41, and overall MolProbity score of 1.13 (51). Molecular structure images were generated using the PyMOL Molecular Graphics System, version 2.0 Schrödinger, LLC.

### Structural modeling

The N and C termini (amino acids 1–9 and 86–88) of full-length SYCE3 were modeled onto the rebuilt SYCE3 structure using MODELLER (52). The SYCE3 domain-swap tetramer was modeled by initial manual positioning of SYCE3 dimers and linking chains via loop sequences in the PyMOL Molecular Graphics System, version 2.0 (Schrödinger, LLC, New York). Constrained-docking was then performed using Rosetta FloppyTail (53), in which SYCE3 dimer folds constituted rigid bodies connected by flexible domain-swap loop sequences. The final model was achieved through iterations of energy minimization using Rosetta Relax (54) interspersed with idealization by PHENIX geometry minimization (49).

### DLS

DLS measurements were recorded at 20 °C on a Malvern Zetasizer Nano S Zen 1600, with protein samples at concentrations of 5–20 mg/ml in 20 mm Tris, pH 8.0, 150 mm KCl, 2 mm DTT in a 3-mm path length quartz cuvette (Hellma). Data were averaged over three replicates, analyzed using the Zetasizer software, and expressed as normalized size distributions per volume.

### Data availability

Rebuilt/re-refined crystallographic atomic coordinates have been deposited in the PDB under accession number 6H86. All other data are available from the corresponding author upon reasonable request.

## Author contributions

O. M. D. performed all experiments. O. R. D. rebuilt and re-refined the SYCE3 crystal structure and built structural models. O. R. D. designed experiments, analyzed data, and wrote the manuscript.

## Supplementary Material

Supporting Information

## Footnotes

3 Please note that the JBC is not responsible for the long-term archiving and maintenance of this site or any other third party hosted site.

## Footnotes

2 The abbreviations used are:

SC: synaptonemal complex
SEC: size-exclusion chromatography
MALS: multi-angle light scattering
SAXS: small-angle X-ray scattering
DLS: dynamic light scattering
PDB: Protein Data Bank.
