# Supplementary material for: A molecular model for self-assembly of the synaptonemal complex protein SYCE3
Source: J Biol Chem. 2019 Apr 25;294(23):9260–75. doi: 10.1074/jbc.RA119.008404 (PMC6556580; doi:10.1074/jbc.RA119.008404)
Supplement: Supporting Information [file supp_294_23_9260__index.html]

A molecular model for self-assembly of the synaptonemal complex protein SYCE3 — Molecular basis of SYCE3 self-assembly — A molecular model for self-assembly of the synaptonemal complex protein SYCE3 — Molecular basis of SYCE3 self-assembly — Supporting Information 

# A molecular model for self-assembly of the synaptonemal complex protein SYCE3

## Supporting Information

- Supporting Information (to be published online) - Supporting Information
