# Supplementary material for: A molecular model for self-assembly of the synaptonemal complex protein SYCE3
Source: J Biol Chem. 2019 Apr 25;294(23):9260–75. doi: 10.1074/jbc.RA119.008404 (PMC6556580; doi:10.1074/jbc.RA119.008404)
Supplement: Supporting Information [file supp_RA119.008404_144259_2_supp_320469_pqj1r5.pdf]

Supporting Information

A molecular model for self-assembly of synaptonemal complex protein SYCE3

Orla M. Dunne[1] and Owen R. Davies[1]

[1]Institute for Cell and Molecular Biosciences, Faculty of Medical Sciences, Newcastle University, Framlington Place, Newcastle upon Tyne NE2 4HH, UK.

To whom correspondence should be addressed:

Owen R. Davies

Institute for Cell and Molecular Biosciences, Faculty of Medical Sciences, Newcastle University, Framlington Place, Newcastle upon Tyne NE2 4HH, UK.

E-mail: owen.davies@newcastle.ac.uk

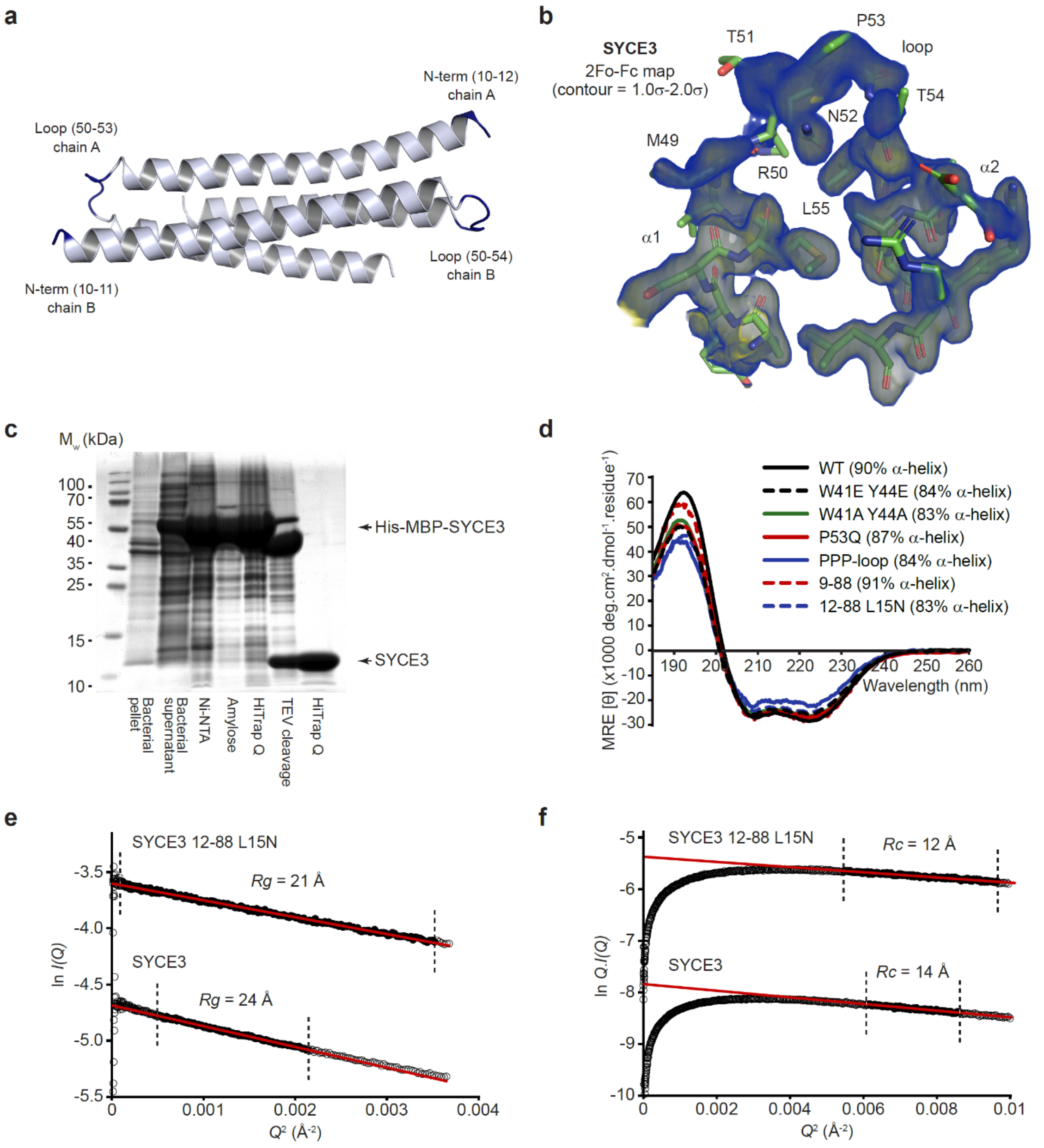

Figure S1

Crystal and solution structures of SYCE3

(**a-b**) The previously reported crystal structure of mouse SYCE3 (PDB accession 4R3Q (31)) was rebuilt and re-refined to include loops and short N-terminal extensions that were missing from the deposited structure. (**a**) SYCE3 structure highlighting the additional residues of the rebuild. (**b**) 2Fo-Fc electron density map shown as a gradient from yellow (2.0σ) to blue (1.0σ) and superimposed on the refined crystallographic model focussed around the rebuilt loop region. (**c**) SDS-PAGE analysis of recombinant

expression and purification of human SYCE3. A recombinant protein harbouring an N-terminal His-MBP tag was expressed in *E. coli* and purified through Ni-NTA, amylose and anion exchange chromatography, followed by TEV cleavage to remove the His-MBP tag, with subsequent anion exchange chromatography. (**d**) Far UV circular dichroism (CD) spectra of SYCE3 constructs (as indicated) recorded between 260 nm and 185 nm in mean residue ellipticity, MRE ([θ]) (x1000 deg.cm^2.dmol^{-1}.residue^{-1}). Data were deconvoluted using the CDSSTR algorithm to determine the relative helical content. (**e**) SAXS Guinier analysis to determine the radius of gyration (*Rg*) of SYCE3 full-length and 12-88 L15N; linear fits are shown in red, with the fitted data range highlighted in black and demarcated by dashed lines. The *Q.Rg* values were < 1.3 and *Rg* was calculated as 24 Å and 21 Å, respectively. (**f**) SAXS Guinier analysis to determine the radius of gyration of the cross-section (*Rc*) of SYCE3 full-length and 12-88 L15N; linear fits are shown in red, with the fitted data range highlighted in black and demarcated by dashed lines. The *Q.Rc* values were < 1.3 and *Rc* was calculated as 14 Å and 12 Å, respectively.

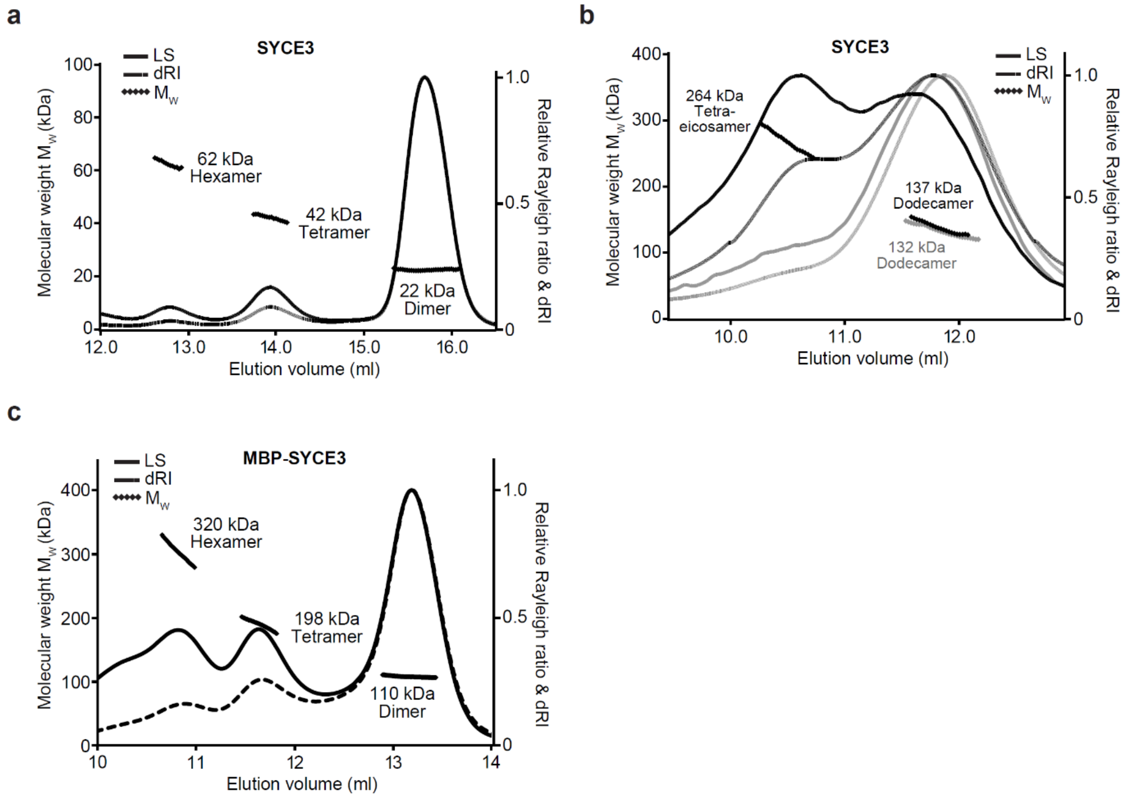

Figure S2

SEC-MALS analysis of SYCE3 self-assembly

(**a-b**) SEC-MALS analysis of SYCE3 following repeated size-exclusion chromatography to enrich for (**a**) low and (**b**) high molecular weight species. (**a**) Analysis following elimination of higher molecular weight species, confirming the presence of the 22 kDa dimer, 42 kDa tetramer and 62 kDa hexamer (theoretical masses – 21 kDa, 43 kDa and 64 kDa). (**b**) Analysis of consecutive size-exclusion chromatography fractions in the higher molecular weight range, highlighting the 132 kDa dodecamer and 264 kDa tetraeicosamer (theoretical masses – 129 kDa and 258 kDa). (**c**) SEC-MALS analysis of MBP-SYCE3, revealing the presence of a 110 kDa dimer, 198 kDa tetramer and 320 kDa hexamer (theoretical masses – 111 kDa, 222 kDa and 334 kDa).

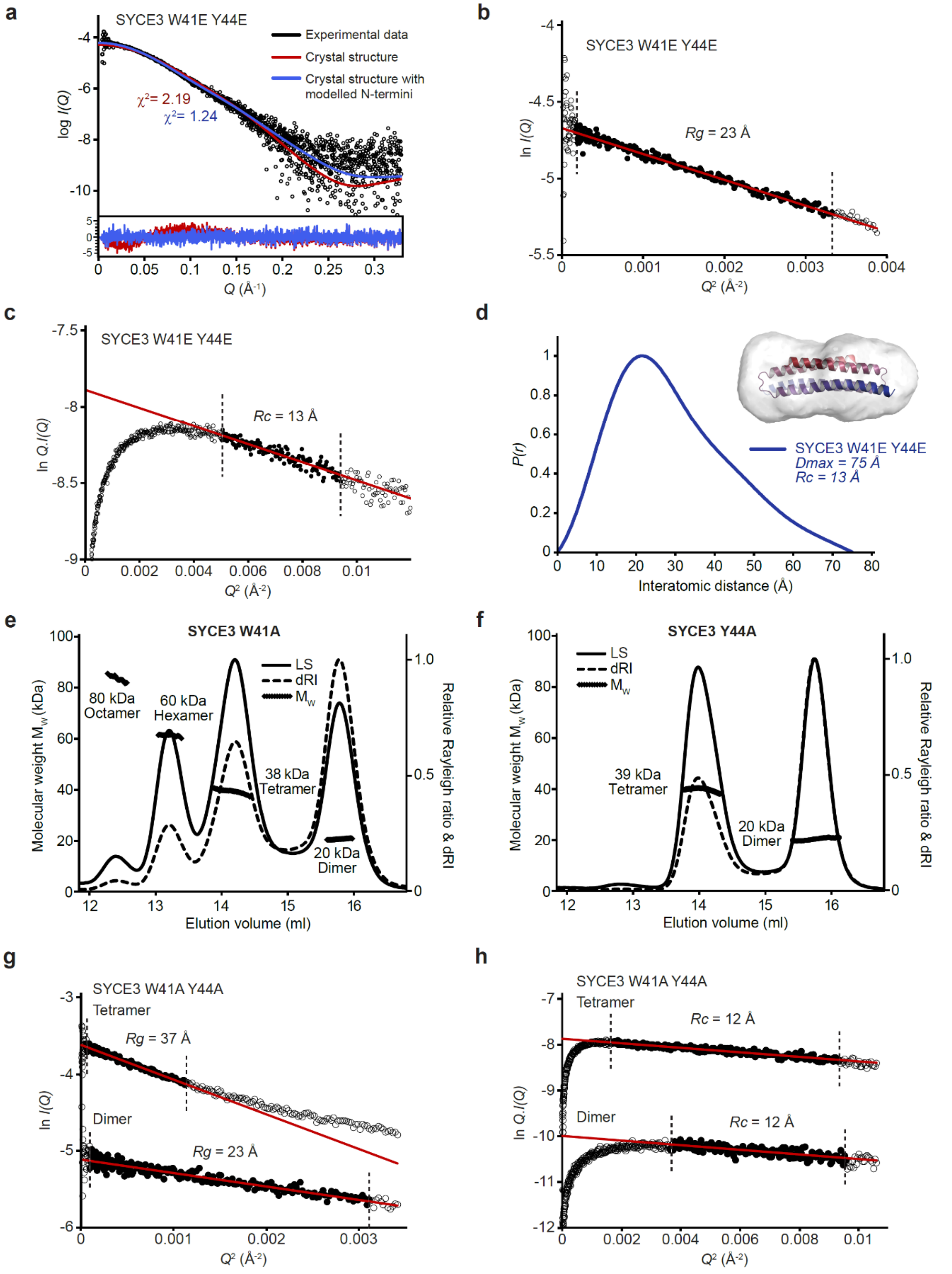

Figure S3

SEC-SAXS and SEC-MALS analysis of SYCE3 W41 and Y44 mutants

(**a-b**) SEC-SAXS analysis of SYCE3 W41E Y44E. (**a**) SAXS scattering data of SYCE3 W41E Y44E overlaid with the theoretical scattering curve of the SYCE3 crystal structure alone (red, χ^2=2.19) and upon the

inclusion of modelled N- and C-termini (blue, χ^2=1.24). Residuals for each fit are shown (inset). (**b**) SAXS Guinier analysis to determine the radius of gyration (*Rg*); linear fits are shown in red, with the fitted data range highlighted in black and demarcated by dashed lines. The *Q.Rg* values were < 1.3 and *Rg* was calculated as 23 Å. (**c**) SAXS Guinier analysis to determine the radius of gyration of the cross-section (*Rc*); linear fits are shown in red, with the fitted data range highlighted in black and demarcated by dashed lines. The *Q.Rc* values were < 1.3 and *Rc* was calculated as 13 Å. (**d**) SAXS *P(r)* interatomic distance distribution of SYCE3 W41E Y44E, showing a maximum dimension of 75 Å. Its SAXS *ab initio* model is shown with the docked crystal structure (NSD = 0.831 ± 0.060; reference model χ^2 = 1.21). (**e-f**) SEC-MALS analysis of SYCE3 W41A and Y44A mutants. (**e**) SYCE3 W41A forms a 20 kDa dimer and decreasing quantities of 38 kDa tetramer, 60 kDa hexamer and 80 kDa octamer (theoretical masses – 21 kDa, 42 kDa, 64 kDa and 85 kDa). (**f**) SYCE3 Y44A forms an approximately 2:1 ratio (by mass) of 20 kDa dimer and 39 kDa tetramer (theoretical masses – 21 kDa and 42 kDa). (**g-h**) SEC-SAXS analysis of SYCE3 W41A Y44A dimer and tetramer species. (**g**) SAXS Guinier analysis to determine the radius of gyration (*Rg*); linear fits are shown in red, with the fitted data range highlighted in black and demarcated by dashed lines. The *Q.Rg* values were < 1.3 and *Rg* was calculated as 23 Å and 37 Å for the dimer and tetramer, respectively. (**h**) SAXS Guinier analysis to determine the radius of gyration of the cross-section (*Rc*); linear fits are shown in red, with the fitted data range highlighted in black and demarcated by dashed lines. The *Q.Rc* values were < 1.3 and *Rc* was calculated as 12 Å for both dimer and tetramer.

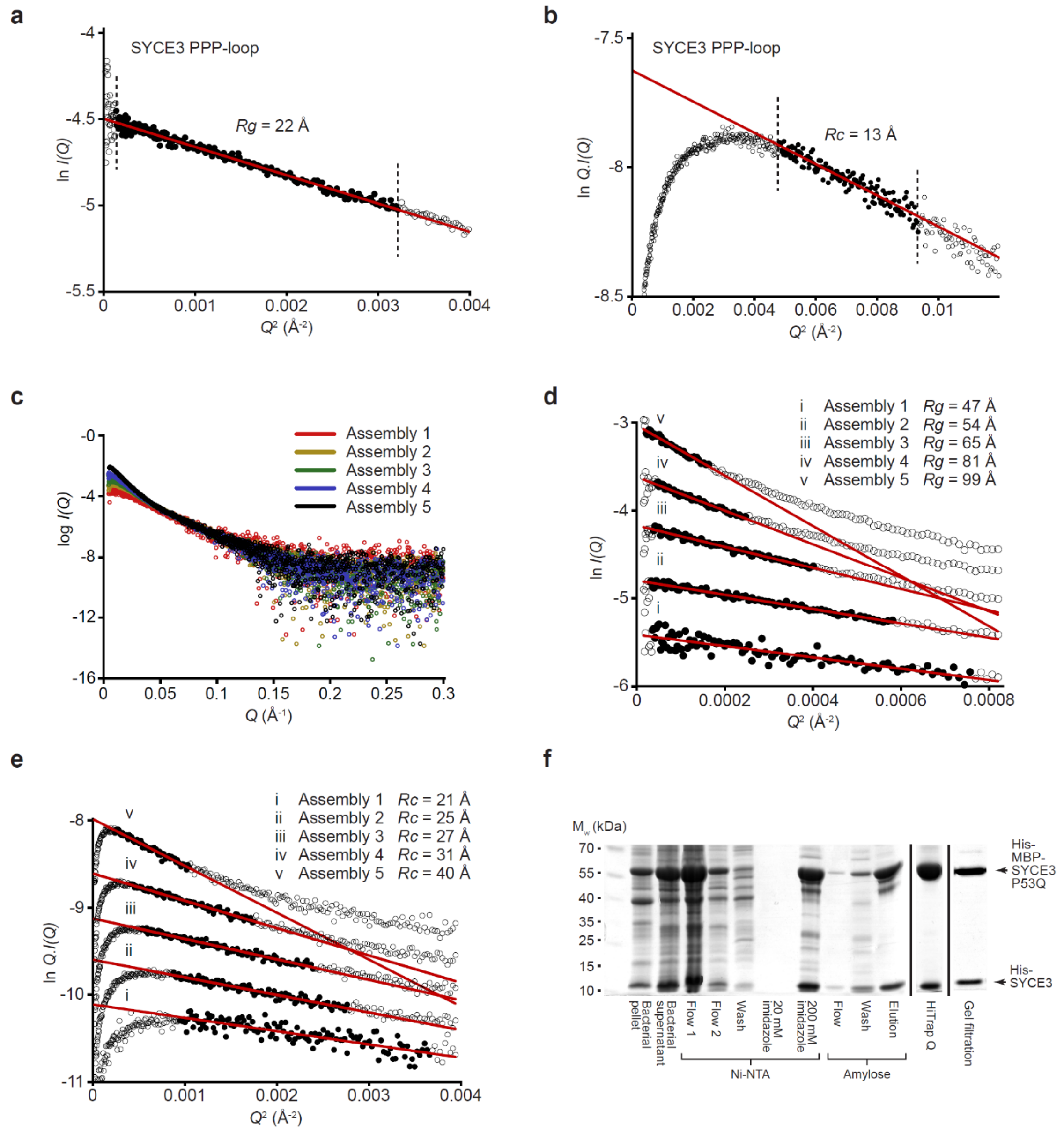

Figure S4

SEC-SAXS analysis of SYCE3 P53Q and PPP-loop mutants

(**a-b**) SEC-SAXS analysis of SYCE3 PPP-loop. (**a**) SAXS Guinier analysis to determine the radius of gyration (*Rg*); linear fits are shown in red, with the fitted data range highlighted in black and demarcated by dashed lines. The *Q.Rg* values were < 1.3 and *Rg* was calculated as 22 Å. (**b**) SAXS Guinier analysis to determine the radius of gyration of the cross-section (*Rc*); linear fits are shown in red, with the fitted data range highlighted in black and demarcated by dashed lines. The *Q.Rc* values were < 1.3 and *Rc* was calculated as 13 Å. (**c-e**) SEC-SAXS analysis of SYCE3 P53Q species (assemblies

1-5 represent the smallest to largest size-exclusion chromatography elution species). (**c**) SAXS scattering curves overlaid and coloured (as indicated) between smallest and largest assemblies. (**d**) SAXS Guinier analysis to determine the radius of gyration (*Rg*); linear fits are shown in red, with the fitted data range highlighted in black and demarcated by dashed lines. The *Q.Rg* values were < 1.3. (**e**) SAXS Guinier analysis to determine the radius of gyration of the cross-section (*Rc*); linear fits are shown in red, with the fitted data range highlighted in black and demarcated by dashed lines. The *Q.Rc* values were < 1.3. (**f**) Co-expression and co-purification of His-MBP-SYCE3 P53Q and His-SYCE3 wild-type through Ni-NTA, amylose, HiTrap Q ion exchange and size-exclusion chromatography. This panel consists of three spliced gels, separated by black vertical lines.

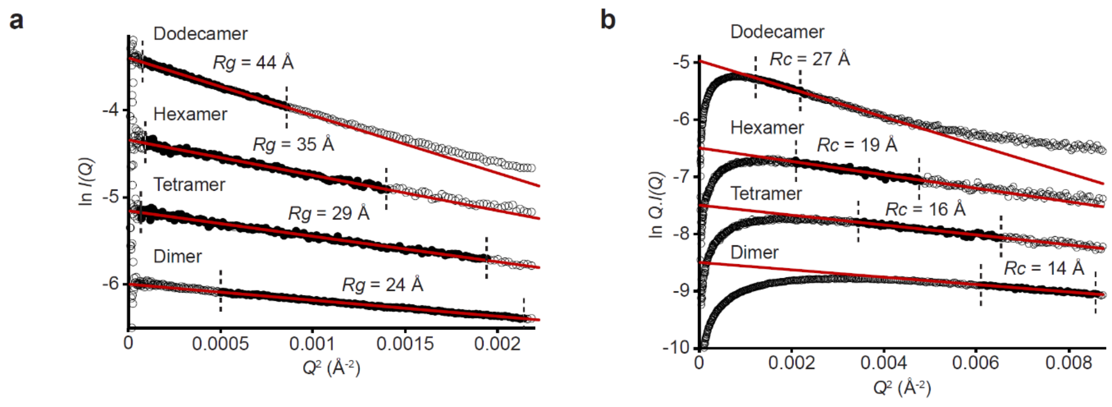

Figure S5

SEC-SAXS analysis of SYCE3 oligomers

(**a-b**) SEC-SAXS analysis of SYCE3 dimer, tetramer, hexamer and dodecamer species. (**a**) SAXS Guinier analysis to determine the radius of gyration (*Rg*); linear fits are shown in red, with the fitted data range highlighted in black and demarcated by dashed lines. The *Q.Rg* values were < 1.3. (**b**) SAXS Guinier analysis to determine the radius of gyration of the cross-section (*Rc*); linear fits are shown in red, with the fitted data range highlighted in black and demarcated by dashed lines. The *Q.Rc* values were < 1.3.
